# The utility of ChatGPT in the assessment of literature on the prevention of migraine: an observational, qualitative study

**DOI:** 10.3389/fneur.2023.1225223

**Published:** 2023-08-17

**Authors:** Leon S. Moskatel, Niushen Zhang

**Affiliations:** Division of Headache and Facial Pain, Department of Neurology, Stanford University, Palo Alto, CA, United States

**Keywords:** artificial intelligence, ChatGPT, migraine, preventive medications, evidence-based medicine

## Abstract

**Background:**

It is not known how large language models, such as ChatGPT, can be applied toward the assessment of the efficacy of medications, including in the prevention of migraine, and how it might support those claims with existing medical evidence.

**Methods:**

We queried ChatGPT-3.5 on the efficacy of 47 medications for the prevention of migraine and then asked it to give citations in support of its assessment. ChatGPT’s evaluations were then compared to their FDA approval status for this indication as well as the American Academy of Neurology 2012 evidence-based guidelines for the prevention of migraine. The citations ChatGPT generated for these evaluations were then assessed to see if they were real papers and if they were relevant to the query.

**Results:**

ChatGPT affirmed that the 14 medications that have either received FDA approval for prevention of migraine or AAN Grade A/B evidence were effective for migraine. Its assessments of the other 33 medications were unreliable including suggesting possible efficacy for four medications that have never been used for the prevention of migraine. Critically, only 33/115 (29%) of the papers ChatGPT cited were real, while 76/115 (66%) were “hallucinated” not real papers and 6/115 (5%) shared the names of real papers but had not real citations.

**Conclusion:**

While ChatGPT produced tailored answers on the efficacy of the queried medications, the results were unreliable and inaccurate because of the overwhelming volume of “hallucinated” articles it generated and cited.

## Introduction

Artificial intelligence (AI) models are poised to synthesize useable outputs, from an ever-expanding medical literature, that physicians and patients can use to inform clinical decisions. For example, an AI model trained on electrocardiograms of migraine patients, with and without aura, was used to suggest that migraine with aura is an independent risk factor for atrial fibrillation (1). A computer based diagnostic engine also performed comparably to specialists in the diagnosis of migraine and probable migraine (2). Large language models, such as the popular Chat Generative Pre-training Transformer (ChatGPT), utilize deep learning algorithms and reinforcement learning from human feedback to generate novel answers to prompts or queries (3). In medicine, ChatGPT has been observed to pass the United States Medical Licensing Examinations (4). Despite the seemingly limitless potential of this technology, one of the biggest flaws of GPT-3 interfaces like Chat GPT is its generation of “hallucinations,” which is an inaccurate response by an AI that is not justified by its training data. For example, in journalism, AI can invent sources, or “hallucinate” articles, including generating not real citations for The Guardian (5). We sought to characterize ChatGPT’s ability to assess the efficacy of medications for the prevention of migraine and to what extent it could support these statements with existing medical literature.

## Methods

This project was exempted from Institutional Review Board Review as it did not meet the criteria for Stanford University’s definition of Human Subject research requiring IRB approval per OHRP 45 CFR 46.102.

We chose the medications to be assessed by aggregating the list of Food and Drug Administration (FDA)-approved medications for the prevention of both episodic and chronic migraine, with the medications addressed in the American Academy of Neurology (AAN) 2012 evidence-based guidelines for the prevention of migraine (6). We elected not to distinguish between prevention of episodic and chronic migraine in order to focus on ChatGPT’s ability to assess the evidence for the treatment of a condition and not on its ability to differentiate treatments for subclassifications of that condition. The triptans approved or recommended for the prevention of menstrually-related migraine were excluded. Medications approved by the FDA after December 30, 2020 were also excluded due to known limitations in ChatGPT’s knowledge of events after 2021. As a negative control, five medications were chosen that had confirmed lack of literature studying their efficacy for migraine, are not used “off-label” for the prevention of migraine, and are not known to work through mechanisms active in the pathophysiology of migraine. In total, this yielded a list of 47 medications.

These 47 medications were assigned to one of six categories: medications that are FDA-approved for the prevention of migraine, medications that are not FDA-approved for the prevention of migraine but have Grade A or B evidence in the 2012 AAN guidelines, medications that are not FDA-approved for the prevention of migraine and have Grade C evidence in the 2012 AAN guidelines, medications that are not FDA-approved for the prevention of migraine and have Grade U evidence in the 2012 AAN guidelines, medications that are not FDA-approved for the prevention of migraine and have Grade A, B, or C *negative* recommendations in the 2012 AAN guidelines, and medications with no role in the prevention of migraine as described above.

OpenAI’s ChatGPT-3.5 was utilized with a new chat session and previous chat history cleared. Each of the 47 medications were queried by one author (LSM) into ChatGPT in one session over 2 days on April 14, 2023 and April 16, 2023 using the phrasing, “is [medication] an effective medication for the prevention of migraine? Give 3 citations if they exist.” ChatGPT’s responses on the efficacy were then manually sorted into five categories, “Yes,” “Some evidence it is effective,” “Limited evidence or research,” “No evidence,” and “Would not give opinion.”

The up to three citations that ChatGPT would give in support of its assessment were then documented and the titles searched on both Google Scholar and PubMed to determine if they were real literature or “hallucinations.” Real papers were then assessed for relevance to the query. These literature search engines were then also surveyed for the first three papers that would result for the search “[medication] migraine prevention.” ChatGPT citations of real papers for each of the medications were then compared to the top three results from the searches of each of Google Scholar and PubMed.

This methodology was determined prior to the initiation of any queries of medications into ChatGPT. This is the primary analysis of these data. There were no missing data as ChatGPT rendered a decision on all 47 medications inputted.

We then assessed ChatGPT’s ability to evaluate the literature through an area under the receiver operating characteristic (ROC) curve, including 95% confidence intervals, for both all medications and the subset of oral medications only. For this, medications in categories 1, 2, and 3 were given the binary outcome “1” denoting their recommendation for the prevention of migraine. Medications in categories 4, 5, and 6 were given the outcome “0” denoting they were not recommended for the prevention of migraine. ChatGPT’s recommendations were then assigned relative strengths of recommendation on a scale of 0 to 1. Answers mapped to “Yes” were assigned “1”; answers mapped to “some evidence it is effective” were assigned “0.66”; “Limited evidence or research as “0.33” and “No evidence” or “would not give opinion” were assigned “0.”

### Statistical analysis

Descriptive statistics were calculated within Microsoft Excel including percentages. Area under the curve for the ROC curve was calculated in STATA, Version 14 (StataCorp LLC).

## Results

The full transcript of the conversations with ChatGPT used to generate the data is presented in Supplemental material S1.

ChatGPT correctly affirmed the efficacy of medications that were either FDA-approved for the prevention of migraine or graded as “Established as effective” or “Probably effective” by the AAN; it deemed all nine FDA-approved medications and all five preventives with Grade A or B evidence effective (Table 1). However, with the medications with Grade C evidence, “Possibly effective,” it only noted that candesartan was effective and stated that the other six medications had limited evidence or research. ChatGPT’s assessment of the 14 medications with Grade U evidence included outcomes from all five of our efficacy categories such as 3/14 stated to be effective and three others reported as having no evidence.

**Table 1:** Summary of queried medications for the prevention of migraine.

Medication	FDA-approved for prevention of migraine	2012 AAN grade of evidence	ChatGPT assessment of efficacy for the prevention of migraine	Number of studies cited by ChatGPT	Number of real studies cited	Number of real and relevant studies cited
Erenumab	Yes	Not applicable	Yes	3	2	2
Fremanezumab	Yes	Not applicable	Yes	3	2	2
Galcanezumab	Yes	Not applicable	Yes	3	3	3
Eptinezumab	Yes	Not applicable	Yes	3	2	2
Propranolol	Yes	A	Yes	3	1	1
Timolol	Yes	A	Yes	3	1	1
Valproic acid	Yes	A	Yes	3	2	2
Topiramate	Yes	A	Yes	3	2	2
OnabotunlinumtoxinA	Yes	A	Yes	3	3	3
Metoprolol	No	A	Yes	3	3	2
Amitriptyline	No	B	Yes	3	2	1
Nadolol	No	B	Yes	3	2	0
Venlafaxine	No	B	Yes	3	1	1
Atenolol	No	B	Yes	3	0	0
Candesartan	No	C	Yes	3	2	2
Lisinopril	No	C	Limited evidence or research	3	0	
Carbamazepine	No	C	Limited evidence or research	3	0	
Nebivolol	No	C	Limited evidence or research	3	0	
Pindolol	No	C	Limited evidence or research	3	0	
Clonidine	No	C	Limited evidence or research	3	0	
Guanfacine	No	C	Limited evidence or research	3	1	0
Gabapentin	No	U	Yes	3	1	0
Verapamil	No	U	Yes	3	2	0
Nicardipine	No	U	Limited evidence or research	No studies cited		
Nifedipine	No	U	Limited evidence or research	No studies cited		
Nimodipine	No	U	Limited evidence or research	No studies cited		
Fluoxetine	No	U	Some evidence it is effective	3	1	0
Fluvoxamine	No	U	Limited evidence or research	3	0	
Protriptyline	No	U	Would not give opinion	3	0	
Acetazolamide	No	U	Yes	3	0	
Cyclandelate	No	U	No evidence	No studies cited		
Acenocoumarol	No	U	No evidence	3	1	0
Coumadin	No	U	Limited evidence or research	3	0	
Picotamide	No	U	No evidence	No studies cited		
Bisoprolol	No	U	Some evidence it is effective	3	1	1
Lamotrigine	No	A negative	Some evidence it is effective	3	3	0
Telmisartan	No	C negative	Limited evidence or research	3	1	1
Clomipramine	No	B negative	Some evidence it is effective	3	0	
Oxcarbazepine	No	C negative	Some evidence it is effective	3	0	
Acebutolol	No	C negative	Limited evidence or research	3	3	0
Clonazepam	No	C negative	Limited evidence or research	3	0	
Nabumetone	No	C negative	Limited evidence or research	3	2	0
Acarbose	No	N/A	No evidence	No studies cited		
Methotrexate	No	N/A	Some evidence it is effective	3	0	
Natalizumab	No	N/A	Some evidence it is effective	3	0	
Famotidine	No	N/A	Limited evidence or research	3	0	
Ceftriaxone	No	N/A	Limited evidence or research	3	0	

2012 AAN Guidelines Grade A evidence denotes “Established as effective,” Grade B denotes “Probably Effective,” Grade C denotes “Possibly effective,” and Grade U denotes “Conflicting or inadequate evidence.” Grade “A negative” denotes “Established as ineffective,” Grade “B negative” denotes “Probably ineffective,” and Grade “C negative” denotes “Possibly ineffective.”

ChatGPT also did not reliably assess medications that have received negative recommendations from the AAN. Of the seven medications the AAN has recommended against with either Grade A, B, or C evidence, 3/7 were reported as having some evidence of efficacy and the other four were reported as having limited evidence or research. Of the five medications that have never been assessed for migraine prevention, only one, acarbose, was correctly stated to have no evidence of its use.

**Figure 1:**
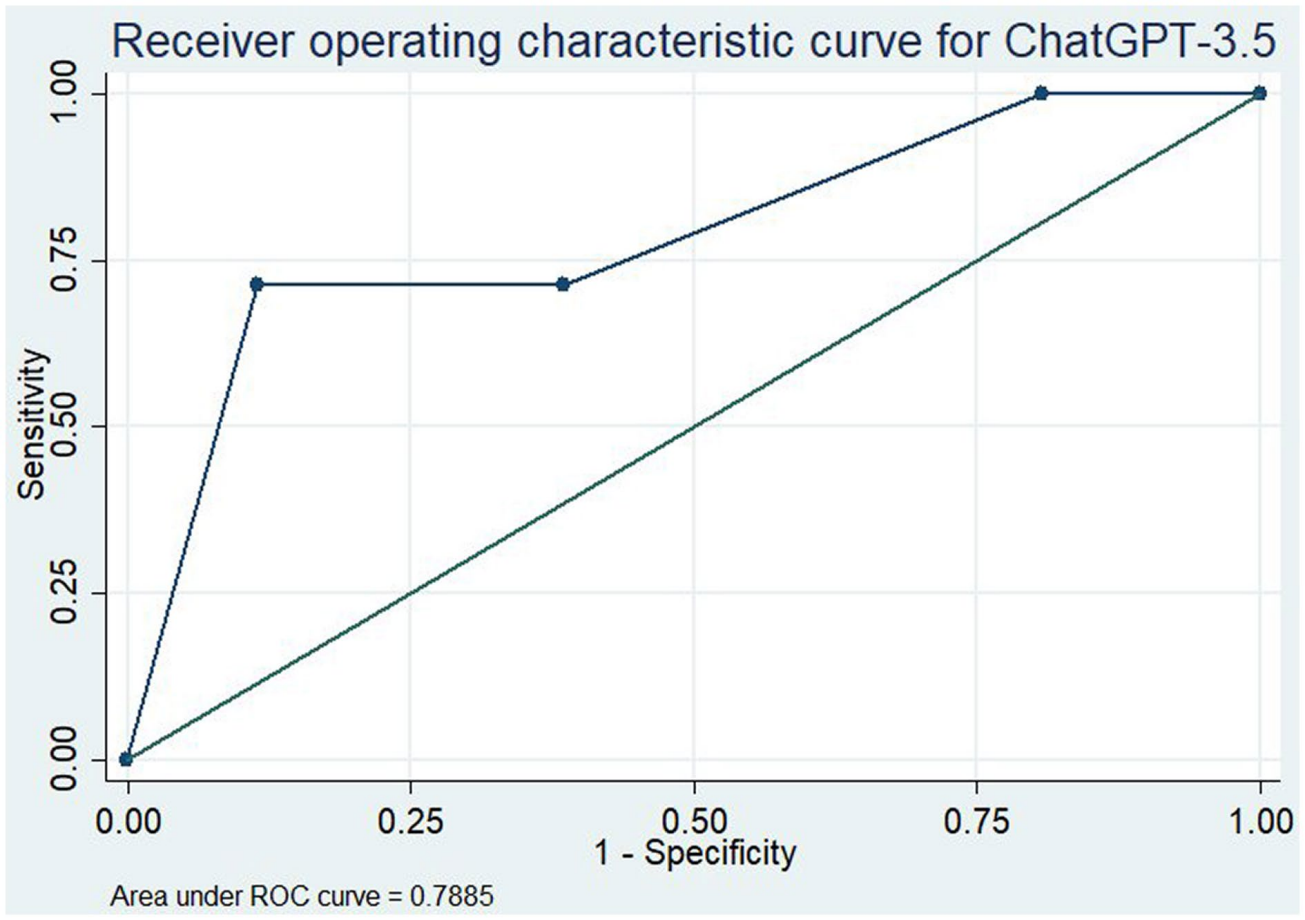
Receiver operating characteristic curve for ChatGPT-3.5 in assessment of the efficacy of all medications for the prevention of migraine.

**Figure 2:**
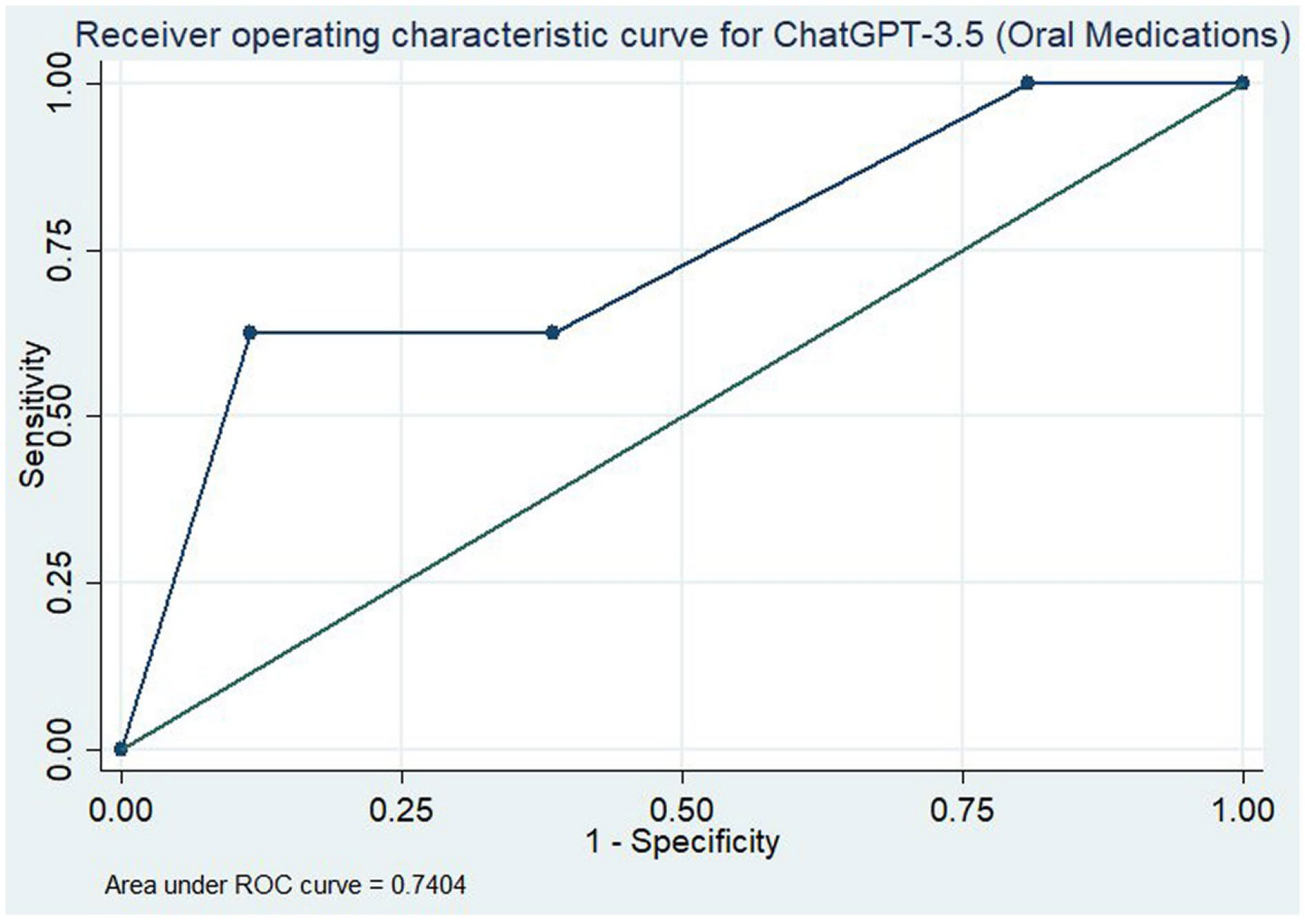
Receiver operating characteristic curve for ChatGPT-3.5 in assessment of the efficacy of only oral medications for the prevention of migraine.

To assess the overall utility of the ChatGPT model for assessment of the evidence, we determined the area under the curve (AUC) for the ROC curve for all medications (Figure 1) and for the oral medications only (Figure 2). With all medications included, this gave an AUC of 0.789 (95% CI: 0.660–0.917). When only oral medications were included, this decreased to an AUC of 0.740 (95% CI: 0.587–0.894).

Concerningly, ChatGPT then predominantly supported its assessments with “hallucinated” papers. Examples of fabricated citations and summaries are presented in Table 2 (The full list of citations for each medication is in Supplementary appendix S1). Of the 47 medications reviewed, ChatGPT generated supporting citations for 41. These 41 papers resulted in 123 cited papers which reduced to 115 papers when duplicates were removed. Of these 115 papers, only 33/115 (29%) were real papers that were correctly cited. Strikingly, 76/115 (66%) papers were ChatGPT “hallucinations” and did not exist. The other 6/115 (5%) papers had the names of real papers but the citation details, including author, journal, and year, were new creations.

**Table 2:** Examples of the “hallucinated” papers generated by ChatGPT and summaries if they were given.

Selected citations of “Hallucinated” papers generated by ChatGPT	Summary of paper by ChatGPT (if given)	Errors in the hallucinated references
Silberstein SD, Neto W, Schmitt J, et al. Topiramate vs. valproate in migraine prophylaxis: a 12-week, randomized, double-blind, double-dummy, parallel-group trial. Cephalalgia. 2007 Sep;27(9):976–87. doi: 10.1111/j.1468-2982.2007.01356.x. PMID: 17661852.	This study compared the efficacy of topiramate and valproic acid for migraine prevention and found that both drugs were similarly effective in reducing the frequency and severity of migraine attacks	Title does not exist DOI does not exist PMID does not exist
Bendtsen L, Jensen R. Clomipramine and migraine revisited: a placebo-controlled study. Cephalalgia. 2000 Oct;20(8):644–50. doi: 10.1046/j.1468-2982.2000.00081.x. PMID: 11028884.	This study evaluated the efficacy of clomipramine in preventing migraines in 39 patients over 16 weeks. The researchers found a significant reduction in migraine frequency and intensity in the clomipramine group compared to placebo	Title does not exist DOI linked to unrelated website PMID linked to non-headache article
Maleki, N., & Becerra, L. (2020). Ceftriaxone as a preventive treatment for migraine: mechanisms of action, potential clinical applications, and future directions. Journal of Headache and Pain, 21(1), 1–10.	None given	Title does not exist
Ribeiro-Filho, N. P., & Toledo, F. L. (2012). Combination of famotidine and ketoprofen in the prophylaxis of migraine without aura: a preliminary study. Arquivos de neuro-psiquiatria, 70(10), 775–778.	This study evaluated the effectiveness of a combination of famotidine and ketoprofen in the prevention of migraines. The study found that the combination therapy significantly reduced the frequency and intensity of migraines	Title does not exist
Tepper SJ, Diener HC, Ashina M, et al. Erenumab in Chronic Migraine: Patient-Reported Outcomes From the Phase 3b LIBERTY Study. Neurology. 2020 Oct 13;95(15):e2115-e2126. doi: 10.1212/WNL.0000000000010642. Epub 2020 Sep 4. PMID: 32887703.	This study examined patient-reported outcomes from the LIBERTY trial, which evaluated erenumab for the prevention of chronic migraine. The study found that erenumab significantly improved patients’ quality of life, reduced their use of acute migraine medications, and reduced the impact of migraines on their daily activities	Title does not exist [but may be a variant of the real “Erenumab in chronic migraine: Patient-reported outcomes in a randomized double-blind study (7)]” DOI linked to unrelated article from *Neurology* PMID linked to non-headache article
Gelfand AA, Thomas KC, Goadsby PJ. Clonidine for the treatment of acute migraine. Headache. 2012;52(3):484–489. doi:10.1111/j.1526-4610.2011.02045.x	None given	Title does not exist DOI linked to unrelated article from *Headache*
Dilli E, Halker R, Vargas B. Protriptyline: A Forgotten Alternative for Migraine Prophylaxis. Headache. 2019 Oct;59(9):1675–1,682. doi: 10.1111/head.13648. Epub 2019 Jul 17. PMID: 31318476.	This study found that protriptyline was effective in reducing the frequency and severity of migraines in patients who had previously failed other migraine preventive medications	Title does not exist DOI linked to unrelated article from *Headache* PMID linked to non-headache article

The propensity for ChatGPT to support its assessment with “hallucinated” papers was investigated further. Seventeen of the 41 medications (41%) for which ChatGPT produced citations were supported by three not real references. Of those with at least one real citation, 9/41 (22%) had one real citation and 10/41 (24%) had two. Only 5/41 (12%) were supported by three real studies. That said, of these 5 medications for which ChatGPT reported three real studies, only two of them, galcanezumab and onabotulinumtoxinA, were supported by three real and relevant studies.

To assess for the relative utility of ChatGPT compared to current literature review standards with the use of Google Scholar and PubMed, we sought to compare the citations generated by ChatGPT to the top three results for a comparable search on those platforms for the medications (Supplementary appendix S2). Of the 24 medications with at least one real citation, six matched at least one citation with Google Scholar, including one that correctly matched two citations, and six that matched one citation with PubMed.

## Discussion

While previous studies and popular media have lauded ChatGPT as a next step in medical information and diagnosis, our study suggests that it is only capable of affirming the efficacy of the medications most widely known and supported for the prevention of migraine and otherwise gave unreliable answers on efficacy of medications; the AUC for both medications overall and for the oral medications overall is not to a sufficient level to be useable when false assessments of the literature could lead to worse outcomes for patients.

Critically, its propensity to “hallucinate” papers to support its reports is concerning, and a known problem with the model (3). Only 34% of the 115 citations produced in our study were real papers, including those with real titles but inaccurate citations, and the other 66% were not real publications. This is particularly worrisome for the negative controls where it produced seemingly credible studies, sometimes with summaries of those studies, for the medications that have no role in the treatment of migraine.

The worrisome nature of the hallucinations is not only due to them being not real, but also due to the highly plausible sounding nature of the citations and summaries, and the use of real names of researchers in the field, such that readers may easily mistake them for real papers. The hallucinated citations are convincing and require thorough literature checks to ensure they are not real. This speaks to the advanced nature of the ChatGPT natural language processing as well as the model’s confidence. ChatGPT never expressed any concern about these being not real citations and so readers, potentially including clinicians and patients, would have no immediate reason to doubt their validity. It is not unimaginable that patients may present to clinic with requests for irrelevant medications to prevent migraine based on conversations with ChatGPT which included “hallucinated” articles.

Journal reviewers and editors, as well as, researchers should be concerned too, as ChatGPT quickly attributed these not real papers to real journals and real authors, similar to previous reports in traditional journalism (5). In our study, 16 not real papers ChatGPT hallucinated received imaginary publication in *Headache* alone and indeed *Headache’s* current editor-in-chief is cited as first author on two of the not real papers. Journals and clinicians may find themselves in difficult positions with inquiries on credible-sounding but non-existent papers attributed to them or their journals. Furthermore, in many of the citations, ChatGPT generated PubMed identifiers (PMID) which are already in use by other real articles. For example, ChatGPT assigned the fictious paper “Chronic Migraine: Patient-Reported Outcomes From the Phase 3b LIBERTY Study” the PMID 32887703, however this PMID is already in use by the 2020 article “COVID-19 and Children With Diabetes-Updates, Unknowns, and Next Steps: First, Do No Extrapolation” (8).

In addition, our interest in comparing the ChatGPT generated citations to those of Google Scholar and PubMed was not realistic because we did not foresee the extent of ChatGPT’s hallucinated articles. Comparatively, searches on Google Scholar and PubMed yielded AAN guidelines and high-yield review papers that could answer the readers’ inquiry, albeit with more effort and provided the reader had access to the paper (6, 9, 10).

Our study is limited by reproducibility. Interactions with ChatGPT are almost by definition, unique and readers are unlikely to get these exact results when attempting to replicate this. Indeed, as ChatGPT is a dynamic and evolving system, as time goes on and additional interactions and information is added to it, the outputs too will change. While the exact results are likely not reproducible, we have included the full transcript of our interaction with ChatGPT to generate our data set for transparency (Supplementary material S1). We also revisited and re-queried a subset of 14 of the medications 2 weeks after the initial data pull to see if different results were obtained and those results were not largely different from those we initially obtained (Supplementary material S2). An additional limitation is that we did not engage in any query or answer revisions or feedback to engineer more accurate ChatGPT answers. This was intentional as we wanted to use phrasing that individuals unfamiliar with the topic may use in their questions; however, it is possible that feedback to ChatGPT on the quality of the responses could generate better results. Moreover, we have used the AAN guidelines as our reference, but using alternate guidelines as the standard, such as those of the Canadian Headache Society or the European Headache Federation, could give different concordance with ChatGPT’s recommendations.

## Conclusion

The role of artificial intelligence in the practice of medicine is rapidly evolving and determining the best usage of these tools is ongoing. While ChatGPT produced tailored answers on the efficacy of the queried medications, the results were unreliable and inaccurate because of the overwhelming volume of “hallucinated” articles it generated and cited. Well-researched and verified medical websites such as UpToDate, WebMD, or MedScape, in addition to traditional online literature search engines including Google Scholar and PubMed should remain as the primary resources for clinicians to determine the optimal treatments for their patients and for patients to learn more about their illness and therapeutic options.

## Data availability statement

The original contributions presented in the study are included in the article/Supplementary material, further inquiries can be directed to the corresponding author.

## Author contributions

LM and NZ: study concept and design, drafting of the manuscript, revising it for intellectual content, and final approval of the completed manuscript. LM: acquisition of data and analysis and interpretation of data. All authors contributed to the article and approved the submitted version.

## Funding

Funding for the open access publication fee was provided by Stanford University’s School of Medicine, Department of Neurology.

## Conflict of interest

The authors declare that the research was conducted in the absence of any commercial or financial relationships that could be construed as a potential conflict of interest.

## Publisher’s note

All claims expressed in this article are solely those of the authors and do not necessarily represent those of their affiliated organizations, or those of the publisher, the editors and the reviewers. Any product that may be evaluated in this article, or claim that may be made by its manufacturer, is not guaranteed or endorsed by the publisher.

## Abbreviations

AI: Artificial intelligence
ChatGPT: Chat generative pre-training transformer
AAN: American academy of neurology
